# New insights on Celtic migration in Hungary and Italy through the analysis of non-metric dental traits

**DOI:** 10.1371/journal.pone.0293090

**Published:** 2023-10-18

**Authors:** Erica Piccirilli, Rita Sorrentino, Federico Lugli, Eugenio Bortolini, Sara Silvestrini, Claudio Cavazzuti, Sara Conti, Szabolcs Czifra, Katalin Gyenesei, Kitti Köhler, Károly Tankó, Antonino Vazzana, Erzsébet Jerem, Anna Cipriani, Antonio Gottarelli, Maria Giovanna Belcastro, Tamás Hajdu, Stefano Benazzi

**Affiliations:** 1 Department of Cultural Heritage, University of Bologna, Ravenna, Italy; 2 Department of Biological, Geological and Environmental Sciences–BiGeA, University of Bologna, Bologna, Italy; 3 Department of Chemical and Geological Science, University of Modena and Reggio Emilia, Modena, Italy; 4 Department of Archaeology and Anthropology, IMF-CSIC (Spanish National Research Centre), Barcelona, Spain; 5 Department of History and Cultures, University of Bologna, Bologna, Italy; 6 National Institute of Archaeology, Hungarian National Museum, Budapest, Hungary; 7 Department of Biological Anthropology, Faculty of Science, Institute of Biology, Eötvös Loránd University, Budapest, Hungary; 8 Hungarian Natural History Museum, Department of Anthropology, Budapest, Hungary; 9 ELKH–ELTE Interdisciplinary Archaeological Research Group, Eötvös Loránd University, Budapest, Hungary; 10 Lamont-Doherty Earth Observatory of Columbia University, Palisades, NY, United States of America; Institute for Anthropological Research, CROATIA

## Abstract

The Iron Age is characterized by an extended interweaving of movements by Celts in Europe. Several waves of Celts from Western and Central Europe migrated southeast and west from the core area of the La Téne culture (between Bourgogne and Bohemia). Through the analysis of non-metric dental traits, this work aims to understand the biological relationship among Celtic groups arrived in Italy and the Carpathian Basin, as well as between local populations and Celtic newcomers. A total of 10 non-metric dental traits were analyzed to evaluate biological affinities among Celts (Sopron-Krautacker and Pilismarót-Basaharc) and Scythians-related populations from Hungary (Tápiószele), Celts from continental Europe (Switzerland and Austria), two Iron Age Etruscan-Celtic sites from northern Italy (Monterenzio Vecchio and Monte Bibele), 13 Iron Age central-southern Italic necropolises, and the northern Italian Bronze Age necropolis of Scalvinetto. Strontium isotopes were measured on individuals from the necropolis of Monte Bibele to infer their local or non-local origin. Results highlight the existence of statistically significant differences between Celts and autochthonous Italian groups. Celtic groups from Hungary and Italy (i.e., non-local individuals of Monterenzio Vecchio and Monte Bibele) share a similar biological background, supporting the historical records mentioning a common origin for Celts migrated to the eastern and southern borders of today’s Europe. The presence of a supposed Steppean ancestry both in Celts from Hungary and Celts from northern Italy corroborates the hypothesis of the existence of a westward migration of individuals and genes from the Steppe towards northern Italy during the Bronze and Iron Age, which contributed to the biological variability of pre-Celtic and later Celtic populations, respectively. Conversely, individuals from central-southern Italy show an autochthonous pre-Iron Age background. Lastly, this work supports the existence of Celtic migratory routes in northern Italy, as shown by biological and cultural admixture between Celts and Italics living together.

## Introduction

The Iron Age Celtic human flow patterns in Europe represent one of the most extensive interweaving of movements of the 1^st^ millennium BC [1]. Moreover, thanks to its crucial placing between Prehistory and History, the Iron Age offers an advantageous point of view to investigate mobility patterns in the past [2]. Therefore, the lifestyle, funerary practices and mobility of Late Iron Age communities identified as Celtic and unified by originally speaking a common Celtic language, nevertheless displaying regional differences [1], have always aroused much interest, bringing archaeologists and anthropologists to build a wide research path in the last years (e.g [3–17]). Particularly, focusing on Celtic mobility patterns, old assumptions of unidirectional large-scale migrations as the cause of the spreading of Celts through Europe have been revised and theories about flows of single individuals or short-distance movements of isolated groups of people are receiving more attention [6, 12, 15–17].

Framed in the Iron Age (1^st^ millennium BC-1^st^ century BC), the peak of the Celtic culture has been traditionally identified with La Tène culture (5^th^ century BC-1^st^ century BC), north of the Alps, in a core region spreading from Bourgogne to Bohemia. Here, a significant cultural change started in the Late Hallstatt period (6^th^ century BC-5^th^ century BC) of the Early Iron Age, and resulted in the emerging La Tène culture in the second half of the 5^th^ century BC [1, 18, 19]. The spreading of distinctive examples of similar material culture and other practices (i.e., language and religion) in different areas of Europe is a notable element in support of the acknowledgment of a common cultural background named commonly “Celtic” [1, 20–24].

Communities of Celtic culture arose and expanded through the European continent, following southern, eastern and western pathways [25]. The ancient Celts reached the Atlantic coast, the Iberian Peninsula and the British Isles in the West, as well as the Carpathian Basin, the Balkans and Asia Minor in the East [1, 13, 26, 27].

**Fig 1:**
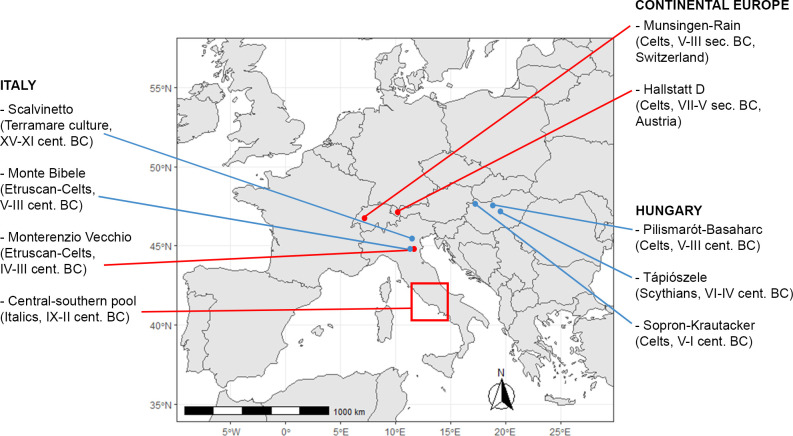
Geographical location of the sites considered in this study. In blue: sites studied here for the first time. In red: sites from the literature.

Despite being now aware that Celtic movements started around the mid-5^th^ century and reached this wide range of territories, the southern and eastern routes have been the main subject of written sources of ancient historians (Ius., XXIV, 4; Tit. Liv., V, 34), documenting that the eastern and southern sides of Europe became the territory of Celtic migrations that reached Italy, on one side, and Pannonia, on the other side, at the beginning of the 4^th^ century BC ([1, 28]; Fig 1).

Celts inhabiting Western and Central Europe north of the Alps moved southwards into Italy with trade purposes, as reported by Plinius the Elder (Plin., XII, 5). In Italy, indeed, Celts could find fertile lands to farm and Mediterranean foods [29–32]. Contacts between the incoming foreigners (i.e., Celts) and the autochthonous Italic people undoubtedly existed even before the so-called great historical Celtic migrations of the 4^th^ century BC. Indeed, in the second half of the 5^th^ century BC already, a phase of infiltration of small groups of Celtic people, or even single families, has been suggested for several sites in northern Italy as a consequence of previous interactions with the autochthonous Etruscans, who had settled into the Po Valley from the end of the 5^th^ century BC. This was the background of the events culminated in the great historical Celtic migrations towards Italy, which brought Celtic people to interact with ancient Italian populations, such as the Etruscans, the Ligures, and the Venetii [29, 30].

Examples of both hostile and peaceful interactions between incoming Celts and autochthonous people of Italy are known especially in some Etruscan communities. For instance, the sites of Marzabotto and Casalecchio di Reno (province of Bologna) were occupied by Celts as a result of harsh conflicts, leading to the demolition of the previous Etruscan reality. On the contrary, the settlements of Monte Bibele and Monterenzio Vecchio (province of Bologna; Fig 1) likely witnessed an integration between Celts and Etruscans, ensuring an interchange of cultural and social aspects and a quiet coexistence [4, 33–35].

Following historical records, the main motivation for the eastwards migration of the Celts in the 4^th^ century BC was overpopulation, leading to scarcity of resources and internal disputes (Ius., XXIV, 4; Tit. Liv., V, 34). There is certainly some truth in Greco-Roman sources mentioning troubles caused by demographic pressure between and/or within Celtic groups, resulting in movements of communities to explore new territories. However, recent studies suggest that many other factors (e.g. climatic changes, economic interests in mineral outcrops) could have facilitated and resulted in flows of people [2, 36]. Numerous archaeological finds support the fact that the Celtic migration to the East reached the western region of the Carpathian Basin (e.g. [37]), where the expansion of the La Tène culture led to the occupation of new territories and the founding of new settlements in the 4^th^ century BC [1, 28, 38]. Unlike reports of ancient authors, archaeological data testify that the early Latènisation in the Carpathian Basin began in the second half of the 5^th^ century. Based on that, it is possible to reconstruct that Celtic communities expanded continuously from West to East along the Danube Valley and through the Vienna Basin to the Carpathian Basin, as well as moving on to the Balkans [39]. Following the eastern expansion routes, two sites from the time of the early occupation of Celts in the Carpathian Basin are studied in this work, because of their significant role in observing the movements of immigrants through this region. The Iron Age necropolis of Sopron-Krautacker is situated near the west-Hungarian border in the eastern Alpine Region, and Pilismarót-Basaharc is located on the bank of the Danube in the central part of the Carpathian Basin. Both of these necropolises are dated between the 5^th^ and 3^rd^/2^nd^ centuries BC ([40, 41]; Fig 1).

Before the arrival of the Celts in northeastern Hungary and the Great Hungarian Plain in the 4^th^-3^rd^ century BC, this area was inhabited by the indigenous population of the Vekerzug culture. This material culture developed from local traditions with the participation of influences from the north Caucasus and eastern influences from the east European forest-steppe in the Early Iron Age [42, 43]. Since 1950, archaeologists have widely investigated the Vekerzug culture and its possible affiliation to the Scythians, namely the Iron Age nomadic populations from the Eurasian steppe (e.g [43–46]). Archaeologists and anthropologists have widely supported that the local Iron Age Vekerzug communities of the central part of the Carpathian Basin, represented in this work by the necropolis of Tápiószele, can be referred to as Scythians-related populations [47–50].

The relatively peaceful contact and co-habitation of the local Vekerzug population with the La Téné communities coming from the West is now demonstrated by archeology with many examples from the mid-3^rd^ century BC [51].

The richness and complexity of the archaeological information regarding the movements of Celts towards Eastern Europe have never been explored biologically, whereas the biological outputs of Celtic flows on the southern route have received relatively more attention in the last years [3, 4, 12].

To better understand the biological aspects of the concept of Celtic migration, this work aims to explore the biological relationship between groups of individuals recognized within the Celtic culture coming from different migratory routes and their potential admixture with autochthonous populations who already inhabited the lands where the incoming Celts settled, through the analysis of non-metric dental traits.

Non-metric dental traits are variations in tooth crown morphology, shape and number of roots, number of teeth, and morphology of specific area of mandibular and maxillary bones [52–54]. Specific subsets of dental phenotypes are considered to be a reliable proxy for gene flow and neutral genetic affinity, and therefore accounting for the genetic background. Several works have assessed biological distance among populations using dental phenotypes as groups of people with similar patterns of dental morphology have a stronger biological relationship than those with differences in dental morphology [5, 6, 52, 54–81].

Therefore, this work aims to assess if individuals from Celtic necropolises from Hungary, Italy, Austria and Switzerland shared a common biological background by showing a stronger affinity to each other than to autochthonous populations of the Italian Peninsula (Bronze Age Terramare Culture individuals and Iron Age Italics) and the Carpathian Basin (Iron Age Scythians-related autochthonous individuals of Tápiószele).

A section of this work will focus on two key sites in northern Italy, Monterenzio Vecchio and Monte Bibele. These two close and coeval cemeteries represent the only example of cultural integration between Etruscans and Celts in Italy [30]. Monterenzio Vecchio has already been investigated using the combination of Sr isotopes and non-metric dental traits to ascertain whether cultural admixture was paralleled by biological admixture and to test the hypothesis of biological integration [4]. Similarly, the individuals from the necropolis of Monte Bibele were distinct in non-locals (likely Celts) and locals (likely Etruscans) by means of Sr isotopes ([3] and this work) following the workflow used for Monterenzio Vecchio [4].

The analysis of Sr isotopes on tooth enamel is a fruitful way to combine geochemistry and archaeology, aiming at investigating provenance and migratory routes of humans in the past. Indeed, tooth enamel registers the bioavailable Sr isotope ratio of the geological area where an individual spent the years of tooth mineralization (i.e. childhood) [82–86].

Ultimately, we aim to explore the interactions of Celtic newcomers with autochthonous inhabitants of the lands they have arrived in. To do that, the Iron age Scythians-related populations from Hungary represented by the necropolis of Tápiószele (late 6^th^ to 4^th^ century BC) is introduced into the study as an example of autochthonous pre-Celtic community in the central part of the Carpathian Basin. On the other hand, for Italy, the Bronze Age northern Italian necropolis of Scalvinetto (1450–1000 BC), attributed to the “Terramare culture” [87], represents an example of autochthonous pre-Celtic community in the Italian Peninsula that will be a proxy to infer about potential biological relationships with the Italic Iron age contexts.

### Archaeological contexts

To investigate the biological distance of Celts from other Iron Age and Bronze Age populations, this study analyzes for the first time non-metric dental traits of individuals from two cemeteries of Celts from Hungary (Sopron-Krautacker, 5^th^-2^nd^ century BC; Pilismarót-Basaharc, late 5^th^-3^st^ century BC), one Scythians-related autochthonous Iron Age necropolis from Hungary (Tápiószele, late 6^th^-4^th^ century BC), the Etruscan-Celtic cemetery of Monte Bibele (late 5^th^-3^rd^ century BC) in Italy, and the Terramare Culture necropolis of Scalvinetto (Middle and Recent Bronze Age, province of Verona). This sample was compared to already published data of the Etruscan-Celtic necropolis of Monterenzio Vecchio (4^th^-3^rd^ century BC) [4], 13 Iron Age Italic sites from central-southern Italy [62] and two continental Celtic core necropolises [5]. Below, the archaeological contexts of the samples used in this study are briefly described.

#### The Celtic necropolises of Sopron-Krautacker and Pilismarót-Basaharc from Hungary

The necropolis and the related settlement of Sopron-Krautacker (Fig 1) are located in the Little Hungarian Plain, northwest of the city of Sopron, at the foot of the Eastern Alps, on the western shore of the Lake Neusiedl [41, 88, 89].

The beginning of the Iron Age settlement and necropolis is dated at the end of Hallstatt D (5^th^ century BC), and it continued to exist and flourish until the Late La Tène period (La Tène D1, i.e., 1^st^ century BC) [36, 41, 88–91].

During the excavations, a large area measuring more than 20.000 square metres and consisting in two cemeteries, the earliest from the Urnfield period (1300–800 BC) and the most recent from the period between the Late Hallstatt and the Middle La Tène, came to light. A total of 57 graves, both cremations and inhumations, belonged to the period of our interest (Late Hallstatt-Middle La Tène). The inhumations included a balanced number of males and females and both juveniles and adults (unpublished data), while the archaeological record comprised many grave goods, both ornamental and weaponry, the latter implying the presence of warriors [38, 88, 89, 91, 92].

The necropolis of Pilismarót-Basaharc (Fig 1) is located in the vicinity of the village Pilismarót, south of the Danube and in the middle section of its bend [40, 93]. The excavations led to the discovery of three different realities: Copper Age, Iron Age, and Avar period cemeteries. Ca. 110 graves, both inhumations and cremations, were dated from the Late Hallstatt to the Middle La Tène Period. Most of the finds were from the Early La Tène, and a minor part has been dated to the Middle La Tène as well as some settlement structures from the Late La Tène period. Ten per cent of the graves were surrounded or covered by stones, and this ritual has been identified more frequently in cremation burials than in the inhumation ones [40, 92–94].

The recent anthropological analysis of a selection of available skeletons showed that both males and females, as well as non-adult and adult age classes, were present, while the archaeological record included a significant presence of knives in the male burials, a scarcity of spearheads and only one sword, the latter being an uncommon feature in warrior graves, differing from the contemporary cemeteries [40].

#### The Scythians-related autochthonous Iron Age necropolis of Tápiószele from Hungary

The necropolis of Tápiószele (Fig 1) is one of the largest cemeteries of the Scythians-related Vekerzug culture in Hungary, and it represents an interesting example of local pre-Celtic community in the Country. It is located near the village of Tápiószele, and dated between the last third of the 6^th^ century and the 4^th^ century BC. The excavation led to the discovery of a total of 455 graves of the Vekerzug culture including 211 cremations and 230 inhumations, plus 13 symbolic burials and one horse grave. Grinding stones and bronze jewels and mirrors were found in female burials and iron knives, spearheads and hatchets were characteristic of male burials, while ceramic grave goods were widely diffused [95–98].

Due to their autochthonous background, the Scythians-related population of Tápiószele can represent a local biological substrate of the Carpathian Basin, later affected by the arrival of Celts.

#### The Terramare necropolis of Scalvinetto from Italy

Scalvinetto (Fig 1) is one of the widest bi-ritual necropolises of the Terramare culture located north of the River Po, today in the territory of Legnano, Province of Verona. The occupation of the related settlement, called Fondo Paviani, covers the period from the Middle Bronze Age 3 to the final Bronze Age 1–2 (1450–1000 BC; [87, 99–101]).

A total of 705 burials were recovered, 437 urn cremations and 268 inhumations, and all of them were dated within the same time span of the settlement [99, 100]. The dating of these burials may be informative of the Bronze Age biological background in northern Italy before the arrival of Celts, and it can be helpful to explore the autochthonous pre-Iron Age substrate and compare it with the biological variability brought by Celtic movements during the Iron Age.

#### The Etruscan-Celtic necropolises of Monterenzio Vecchio and Monte Bibele from Italy

The necropolis of Monterenzio Vecchio (Fig 1) is placed 30 km south-east of Bologna, along the eastern hill side of the Idice river valley, in the Tuscan-Emilian Apennines. The excavation brought to the discovery of a total number of 50 graves, representing a population of males, females, and non-adults. Inhumation is much more frequent than cremation, which is represented by only four burials. The analyzed grave goods, such as Mediterranean banquet items, transalpine and Italic weapons, allowed to chronologically assign the graves to the La Tène B2 phase (4^th^-3^rd^ century BC) and to detect the presence of both local and non-local individuals buried, suggesting interaction between autochthonous Etruscans and Celtic immigrants [4, 102–104].

The necropolis of Monte Tamburino at Monte Bibele (Fig 1) is dated between the end of the 5^th^ and the 3^rd^ century BC, and placed close to the necropolis of Monterenzio Vecchio, on the top of Monte Tamburino, one of the three peaks that constitute the mountain range of Monte Bibele [30, 105–108]. The excavation campaigns brought to the discovery of 161 tombs, nevertheless at least five burials, and maybe more, were found with illegal excavations. Due to that, the total number of graves is about 170 units, dating from the end of the 5^th^ to the 3^rd^ century BC and representing the largest Celtic context in Italy. Strong similarities are detectable with the funerary context of Monterenzio Vecchio, for example the higher presence of inhumations than cremations and the mixing of local banquet-related (Etruscan like) and non-local war-related grave goods [30, 106, 108].

#### The Italic Iron Age necropolises from central-southern Italy

The comparative Italic sample from Coppa and colleagues comes from 13 Iron Age samples in central-southern Italy (Fig 1), dated in the time span between the 9^th^ and the 2^nd^ century BC, which can be informative of the biological variability of populations who are not known to have encountered and admixed with Celtic immigrants during Iron Age [62]. In particular, the 13 Iron Age Italic sites from central-southern Italy have been grouped in three periods by the authors: A (early 9^th^-8^th^ century BC), B (middle 7^th^-5^th^ century BC), and C (late 4^th^-2^nd^ century BC), and the sample consists in Etruscans (ETB, ETC), Latins (LAA, LAB, LAC), Picentes (PCB, PCC), Montani (MON), Sulmona (SUL), Samnites (SAM), Campani (CAA, CAB, CAC) [62].

#### The Celtic necropolises from continental and non-continental Europe

The comparative Celtic sample from Anctil is composed by continental proto-Celtic individuals (7^th^-5^th^ century BC, named HalD) from Hallstatt (Austria) and continental Munsingen-Rain (5^th^-3^rd^ century BC, named MunRain) from Munsingen (Switzerland), taken as examples of core regions Celtic necropolises ([5]; Fig 1).

Hallstatt D represents the site where the first material culture associated with the early Celtic culture was discovered and identified. Munsingen-Rain consists of individuals dated to La Tène A, B, C periods [5].

## Materials and methods

### Sample

Samples of teeth, alveolar bones and maxillary and mandibular bones from a total of 1294 individuals have been included in this study (Table 1). Novel data were collected from individuals from Sopron-Krautacker, Pilismarót-Basaharc, Tápiószele, Scalvinetto and Monte Bibele (S1 Table, in which specimen numbers, sex, age at death and repository information are also provided). Collections were accessed with permission from the relevant curators.

**Table 1:** Samples used for non-metrical dental trait analysis.

Group	Sample	Abbreviation	Chronological periods	N
Celts from Hungary	Sopron-Krautacker*	SK	5^th^ to 1^st^ century BC	10
	Pilismarót-Basaharc*	PB	5^th^ to 3^rd^ century BC	19
Scythians-related populations from Hungary	Tápiószele*	Táp	Late 6^th^ to 4^th^ century BC	15
Italian Terramare individuals (northern Italy)	Scalvinetto*	Sca	1450 to 1000 BC	11
Italian Etruscans-Celts (northern Italy)	Monte Bibele locals (likely Etruscans)*	MBL	Late 5^th^ to 3^rd^ century BC	32
	Monte Bibele non-locals (likely Celts)*	MBNL	Late 5^th^ to 3^rd^ century BC	12
	Monterenzio Vecchio locals (likely Etruscans)^1^	MVL	4^th^ to 3^rd^ century BC	8
	Monterenzio Vecchio non-locals (likely Celts)^1^	MVNL	4^th^ to 3^rd^ century BC	10
Italics (central-southern Italy)	Ancient Etruscans^2^	ETB	Middle 7^th^ to 5^th^ century BC	95
	Recent Etruscans^2^	ETC	Late 4^th^ to 2^nd^ century BC	102
	Archaic Latini^2^	LAA	Early 9^th^ to 8^th^ century BC	33
	Ancient Latini^2^	LAB	Middle 7^th^ to 5^th^ century BC	41
	Recent Latini^2^	LAC	Late 4^th^ to 2^nd^ century BC	94
	Ancient Piceni^2^	PCB	Middle 7^th^ to 5^th^ century BC	136
	Recent Piceni^2^	PCC	Late 4^th^ to 2^nd^ century BC	211
	Montani^2^	MON	Early 9^th^ to 8^th^ century BC/B	40
	Sulmona^2^	SUL	Late 4^th^ to 2^nd^ century BC	52
	Samnites^2^	SAM	Middle 7^th^ to 5^th^ century BC	163
	Archaic Campani^2^	CAA	Early 9^th^ to 8^th^ century BC	65
	Ancient Campani^2^	CAB	Middle 7^th^ to 5^th^ century BC	28
	Recent Campani^2^	CAC	Late 4^th^ to 2^nd^ century BC	54
Celts from continental Europe	Hallstatt D^3^	HalD	7^th^ to 5^th^ century BC	30
	Munsingen-Rain^3^	MunRain	5^th^ to 3^rd^ century BC	33
	**Total**			**1294**

*Analyzed in this study.

^1^From Sorrentino et al., 2018 [4].

^2^From Coppa et al., 1998 [62].

^3^From Anctil, 2016 [5].

Before performing the non-metric dental traits analysis, information about sex and age of the individuals were collected.

Skeletal samples from Scalvinetto were examined in the Bones Lab, Department of Cultural Heritage (Ravenna), University of Bologna, by E.P. Sex determination was conducted primarily on cranial morphology and pelvic morphology. When these two districts were not well preserved, a metric analysis of clavicle, humerus and femur was performed. Age at death estimation was conducted through a series of methods, and the standards published by Buikstra and Ubelaker were used to assign the individuals to the proper age cohorts [109–125].

Sex and age-related unpublished data from the individuals of Sopron-Krautacker, Pilismarót-Basaharc and Tápiószele were provided by Sz.Cz., K.G., K.K, and T.H.

Sex determination and age at death estimation of the individuals from Monte Bibele were taken from the existing literature [108].

Non-metric dental traits data from the necropolis of Monterenzio Vecchio, continental Celts, and central-southern Italic individuals are from the existing literature [4, 5, 62].

### Strontium isotopes

Strontium isotopes were used to assess potential local and non-local individuals in the two necropolises of Monterenzio Vecchio and Monte Bibele (Bologna, Italy) for which coexistence between Celts and Etruscans has been suggested [30]. While data are already available for Monterenzio Vecchio [4], only 21 individuals of Monte Bibele were already analyzed for strontium isotopes by Scheeres and colleagues [3]. Thus, to increase the sample size of locals and non-local individuals of Monte Bibele group, a total of 38 teeth/individuals were analyzed for strontium isotopes here.

Strontium isotopes have been extensively used to determine the provenance of individuals, as ^87^Sr/^86^Sr ratio in skeletal tissues is linked to that of the living location. Particularly, the tooth enamel preserves ^87^Sr/^86^Sr ratio of the location where the individuals lived during the age of mineralization [82]. In this study, we compare the ^87^Sr/^86^Sr of tooth enamel of available teeth that mineralized first to discriminate local and non-local individuals in Monte Bibele. When possible, we selected first molars (N = 28), but when the first molar was not available, we selected in the order of priority the second molar (N = 2), the first premolar (N = 4), and the second premolar (N = 4), accounting for infancy and early childhood [126].

Strontium isotopes analyses have been performed at the Geochemistry Lab of the Department of Chemical and Geological Science (UNIMORE), following the protocol of [84]. Specifically, ca. 5 mg of enamel (n = 38) were digested with suprapur nitric acid and processed through ion exchange chromatography. Sr was separated using 30 μl columns [127] filled with Eichrom Sr-spec resin. The ^87^Sr/^86^Sr ratio was determined with a Neptune MC-ICPMS housed at the Centro Interdipartimentale Grandi Strumenti (UNIMORE). Analytical details are reported in [85]. The Sr isotope ratio of the samples was normalized to an accepted NIST-SRM987 value of 0.710248. Repeated analyses of the NIST-SRM987 provided a mean ^87^Sr/^86^Sr ratio of 0.710236 ± 0.000013 (2 SD, n = 13).

The local Sr isotope baseline of Monte Bibele was determined by combining the local Sr isotope signature of Monterenzio Vecchio [4] with those already available for Monte Bibele [3], resulting in a local range of 0.7086 and 0.7091, compatible with the bioavailable Sr isotope range calculated from the Italian isoscape [86] in a radius of 15 km from the site (0.7085–0.7090). Such an interval agrees with the expected Sr isotopic ratio of sedimentary rocks outcropping in the area, mostly dating to the Miocene-Pleistocene [3, 128]. We acknowledge that this baseline may over-/underestimate the number of individuals identified as non-locals, however, we decided to be consistent with previous works [3, 4] rather than use a different local range.

### Non-metric dental trait recording and analysis

The assessment of phenotypic variability of non-metric dental traits was used as a proxy for measuring gene flow, and consequently biological relationships between populations [55, 76, 77]. Dental and oral osseous traits observed in individuals included in the analysis (Table 1) were recorded through a scale of numerical degrees of expression of the given morphological variant, following the Arizona State University Dental Anthropology System (ASUDAS) [129].

Initially, a total of 42 discrete dental and oral osseous traits were collected for each individual of Monte Bibele, Sopron-Krautacker, Pilismarót-Basaharc, Tápiószele and Scalvinetto, but only 10 traits offered comparability with the sample coming from different sources [4, 5, 62]; Table 2). Among these 10 non-metric dental traits, 7 are comprised in one of the 267 traits combinations proposed by Rathmann and colleagues [55] as the most informative on neutral genetic affinities. The higher degree of each trait was recorded according to ASUDAS rank-scales of occurrence [129], and then expression of traits was dichotomized into “absent” or “present” using threshold breakpoints taken from the comparative literature and finally transformed into frequencies [4, 5, 62]; Table 2). Threshold breakpoints used for dichotomization are showed in Table 2. An initial statistical test (i.e., Pearson’s product-moment coefficient of correlation (r)) was used on the selected 10 traits to ascertain for pairwise dependence between them and sex of the novel data, with the aim to operate a preliminary cleaning of the data. Then, the main statistical analyses were performed. Using the R package AnthropMMD v.4.1.3, the Mean Measure of Divergence (MMD) with the Freeman and Tukey correction on trait frequencies was carried out to calculate biological divergence between each sample pair [66, 67, 130–132]. MMD allows to measure the biodistance between samples as it mathematically produces high values when the samples are biologically distant and lower values when they are similar. MMD values depict significant biological distance (*p* <0.025) among samples when values for MMD are higher than 2 standard deviations [130–133]. In addition to MMD, also the overall Measure of Divergence (MD) was calculated. MD is the sum over all the possible couples of samples for a given trait, and it works as an indicator of the utility of the given traits in the MMD analysis [131]. The thus obtained MMD pairwise distance matrix was used to visually explore biological distances through metric multidimensional scaling (MDS) and hierarchical cluster analysis (Ward’s method). Code and data analysis were made in R version 4.1.3 [134].

**Table 2:** Percentages (%) of discrete dental traits and number of individuals (n) in the samples.

Traits	* *	SK	PB	Táp	Sca	MBL	MBNL	MVL	MVNL	MunRain	HalD	LAA	LAB	LAC	ETB	ETC	PCB	PCC	CAA	CAB	CAC	SUL	SAN	MON
Double shoveling UI1	*%*	57.1	27.3	66,7	0.0	8.3	0.0	0.0	20.0	0.0	0.0	12.5	0.0	0.0	16.7	9.1	15.6	0.0	14.3	0.0	4.8	8.3	3.3	0.0
(+ = ASU 2–6)	*n*	7	11	3	6	12	6	7	10	33	30	8	5	19	6	11	32	41	14	7	21	12	30	9
Tuberculum dentale UI2	*%*	85.7	30.8	62,5	62.5	16.7	0.0	57.1	44.4	60.6	30.0	66.7	42.9	65.4	50.0	43.5	62.2	57.5	65.0	53.8	24.0	60.0	50.0	58.8
(+ = ASU 2–6)	*n*	7	13	8	8	12	5	7	9	33	30	9	7	26	10	23	45	80	20	13	25	10	50	17
Distal accessory ridge UC	*%*	33.3	18.2	0.0	50.0	62.5	33.3	33.3	20.0	57.6	33.3	33.3	62.5	70.4	81.8	58.3	73.0	66.1	92.9	60.0	66.7	63.6	74.3	57.1
(+ = ASU 2–5)	*n*	6	11	5	6	16	3	3	5	33	30	9	8	27	11	12	37	59	14	5	15	11	35	14
Carabelli’s trait UM1	*%*	16.7	23,5	28,6	66.7	53.8	66.7	71.4	25.0	24.2	10.0	71.4	62.5	78.6	58.2	50.0	58.2	62.8	70.8	53.8	48.3	37.5	71.7	63.2
(+ = ASU 2–7)	*n*	6	17	7	9	13	3	7	8	33	30	14	16	42	12	20	55	94	24	13	29	16	60	19
Parastyle UM3	*%*	0.0	0.0	20.0	11.1	9.1	40.0	0.0	40.0	27.3	20.0	40.0	6.7	10.0	0.0	10.0	23.8	20.3	30.0	9.1	13.8	0.0	8.2	0.0
(+ = ASU 1–5)	*n*	2	14	5	9	11	5	7	5	33	30	10	15	40	18	20	42	69	20	11	29	12	49	20
Premolar root number UP1	*%*	33.3	12.5	0.0	50.0	41.7	50.0	60.0	37.5	27.3	43.3	50.0	60.0	0.0	73.3	57.1	63.6	69.8	47.1	81.8	47.4	63.6	78.3	52.6
(+ = ASU 2+)	*n*	6	8	3	6	12	2	5	8	33	30	6	10	2	15	28	55	86	17	11	19	22	46	19
Molar root number UM2	*%*	100.0	100	50.0	57.1	64.7	60.0	100.0	88.9	30.3	63.3	70.0	62.5	75.0	73.7	69.6	72.9	69.1	84.2	84.2	69.2	44.4	73.5	61.1
(+ = ASU 3+)	*n*	4	5	4	7	17	5	7	9	33	30	10	8	4	19	23	48	81	19	19	13	18	49	18
Lingual Cusp LP2	*%*	85.7	78.6	100.0	85.7	81.0	100.0	75.0	77.8	27.3	23.3	50.0	41.7	54.3	24.0	39.4	43.8	46.2	65.0	58.3	37.8	58.3	62.7	36.8
(+ = ASU 2–9)	*n*	7	14	11	7	21	8	8	9	33	30	12	12	35	25	33	48	65	20	12	37	12	59	19
Groove Pattern LM2	*%*	33.3	16.7	27,3	25.0	7.1	37.5	12.5	0.0	0.0	36.7	26.7	44.4	27.8	33.3	19.6	28.1	23.6	39.4	23.5	15.2	13.6	12.7	16.0
(+ = ASU Y)	*n*	9	18	11	8	14	8	8	5	33	30	15	18	36	36	51	64	110	33	17	33	22	63	25
Cusp Number LM1	*%*	0.0	0.0	0.0	25.0	15.4	0.0	0.0	0.0	0.0	16.7	0.0	6.2	8.1	3.2	2.2	2.3	3.2	12.1	0.0	0.0	9.1	1.4	0.0
(+ = ASU 6+)	*n*	10	18	9	4	13	7	8	9	33	30	13	14	34	31	45	44	93	33	16	25	22	69	23

## Results

### Strontium isotopes for Monte Bibele

**Fig 2:**
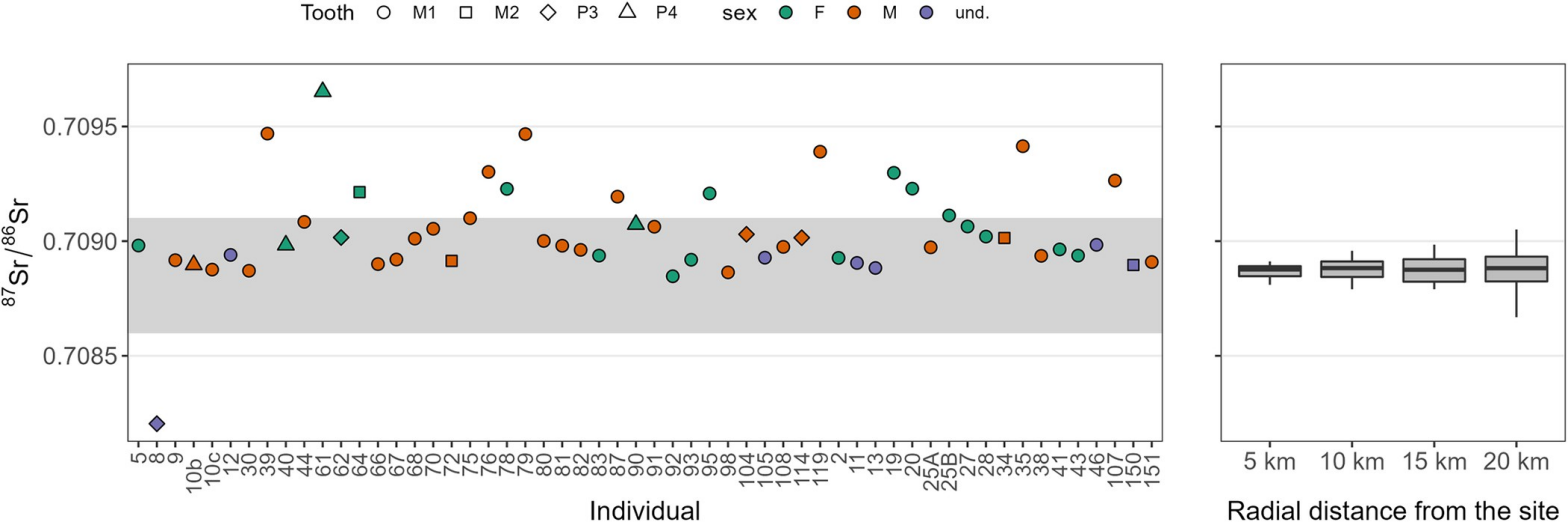
Sr isotope results. (Left panel) Sr isotope data for the individuals considered in this study; the grey area is the likely local baseline; (right panel) Sr isotope signature of the Italian isoscape, resampled at a radial distance of 5, 10, 15 and 20 km from the site.

Strontium isotopes for individuals of Monte Bibele are showed in Table 3 and Fig 2, including previous data from Scheeres and colleagues [3]. Not all the individuals analyzed for strontium isotopes were included in non-metrical dental trait analysis. In fact, 13 individuals (MB8, MB10b, MB20, MB25b, MB40, MB61, MB92, MB93, MB98, MB104, MB114, MB119, MB150) were not considered due to the low number of teeth preserved and/or too many missing data (Table 3). Of the total number of individuals (n = 59), n = 40 individuals yielded ^87^Sr/^86^Sr ratios ranging between 0.7091 and 0.7088, falling in the range of the local Sr isotope signature, thus suggesting that these individuals were likely born in Monte Bibele. Of the local individuals, n = 32 were included in non-metrical dental trait analysis. N = 16 individuals yielded ^87^Sr/^86^Sr ratios ranging from 0.7096 and 0.7091, non-compatible with the local range thus possibly indicating a non-local provenance, and n = 12 of them were included in non-metrical dental traits analysis. One individual (MB8) has ^87^Sr/^86^Sr value of 0.7082, likely indicative of an additional non-local signature as already observed for one individual of Monterenzio Vecchio (MV21; [4]). However, it was not possible to include MB8 in non-metrical dental traits analysis.

**Table 3:** ^87^Sr/^86^Sr ratio of the individuals from Monte Bibele.

**Individual**	**Tooth sampled**	**^87^Sr/^86^Sr**	**±2σ**	**Sex**	**Local/ Non-local**	**NMDT** *
5	LLM1	0.70898	0.00001	F	L	yes
8	LLP3	0.70820	0.00001	-	NL	no
9	LLM1	0.70892	0.00001	M	L	yes
10b	URP4	0.70890	0.00001	M	L	no
10c	ULM1	0.70888	0.00001	M	L	yes
12	URM1	0.70894	0.00001	-	L	yes
30	ULM1	0.70887	0.00001	M	L	yes
39	LRM1	0.70947	0.00001	M	NL	yes
40	LLP4	0.70898	0.00001	F	L	no
44	LRM1	0.70908	0.00001	M	L	yes
61	UP4	0.70965	0.00001	F	NL	no
62	LRP3	0.70902	0.00001	F	L	yes
64	ULM2	0.70921	0.00001	F	NL	yes
66	ULM1	0.70890	0.00001	M	L	yes
67	ULM1	0.70892	0.00001	M	L	yes
68	LRM1	0.70901	0.00001	M	L	yes
70	URM1	0.70905	0.00001	M	L	yes
72	LRM2	0.70891	0.00001	M	L	yes
75	LLM1	0.70910	0.00001	M	NL	yes
76	LLM1	0.70930	0.00001	M	NL	yes
78	LRM1	0.70923	0.00001	F	NL	yes
79	LRM1	0.70947	0.00001	M	NL	yes
80	LLM1	0.70900	0.00001	M	L	yes
81	LLM1	0.70898	0.00001	M	L	yes
82	URM1	0.70896	0.00001	M	L	yes
83	LRM1	0.70894	0.00001	F	L	yes
87	ULM1	0.70919	0.00001	M	NL	yes
90	LRP4	0.70907	0.00001	F	L	yes
91	LRM1	0.70906	0.00001	M	L	yes
92	ULM1	0.70885	0.00001	F	L	no
93	ULM1	0.70892	0.00001	F	L	no
95	LRM1	0.70921	0.00001	F	NL	yes
98	ULM1	0.70886	0.00001	M	L	no
104	URP3	0.70903	0.00001	M	L	no
105	URM1	0.70893	0.00001	-	L	yes
108	LRM1	0.70898	0.00001	M	L	yes
114	LRP3	0,70902	0.00001	M	L	no
119	LRM1	0.70939	0.00001	M	NL	no
	**From Scheeres et al., 2013**					
**Individual**	**Tooth sampled**	^**87**^ **Sr/** ^**86**^ **Sr**	**±2σ**	**Sex**	**Local/ Non-local**	NMDT*
2	LLM1	0.70893	0.00001	F	L	yes
11	LRM1	0.70891	0.00001	-	L	yes
13	URM1	0.70888	0.00001	-	L	yes
19	LRM1	0.7093	0.00001	F	NL	yes
20	LLM1	0.70923	0.00001	F	NL	no
25a	LRM1	0.70897	0.00001	M	L	yes
25b	LLM1	0.70911	0.00001	F	NL	no
27	LLM1	0.70906	0.00001	F	L	yes
28	LRM1	0.70902	0.00001	F	L	yes
34	LRM2	0.70901	0.00001	M	L	yes
35	URM1	0.70941	0.00001	M	NL	yes
38	LRM1	0.70894	0.00001	M	L	yes
41	ULM1	0.70896	0.00001	F	L	yes
43	LRM1	0.70894	0.00001	F	L	yes
46	LRM1	0.70898	0.00001	-	L	yes
107	LRM1	0.70926	0.00001	M	NL	yes
150	LRM2	0.7089	0.00001	-	L	no
151	LLM1	0.70891	0.00001	M	L	yes

*Individuals analyzed for non-metric dental traits (NMDT).

### Biodistance

The 10 selected trait frequencies are listed in Table 2. The original set of 42 trait frequencies in the newly analyzed necropolises is shown in S2 Table. On the 10 traits selected, a correlation test using Pearson’s product-moment correlation coefficient found that no traits were significantly correlated (*p* < 0.05) with each other for the novel data, except for Groove pattern LM2 and Double shoveling UI1 although with a moderate correlation (r = 0.43). Sex moderately correlates with Double shoveling UI1 (r = -0.42; *p*-value = 0.02) and Parastyle UM3 (r = 0.34; *p*-value = 0.04). Since the correlation not exceeded 0.05 in each case (S1 Fig), these traits were maintained so the dataset was not reduced anymore.

**Fig 3:**
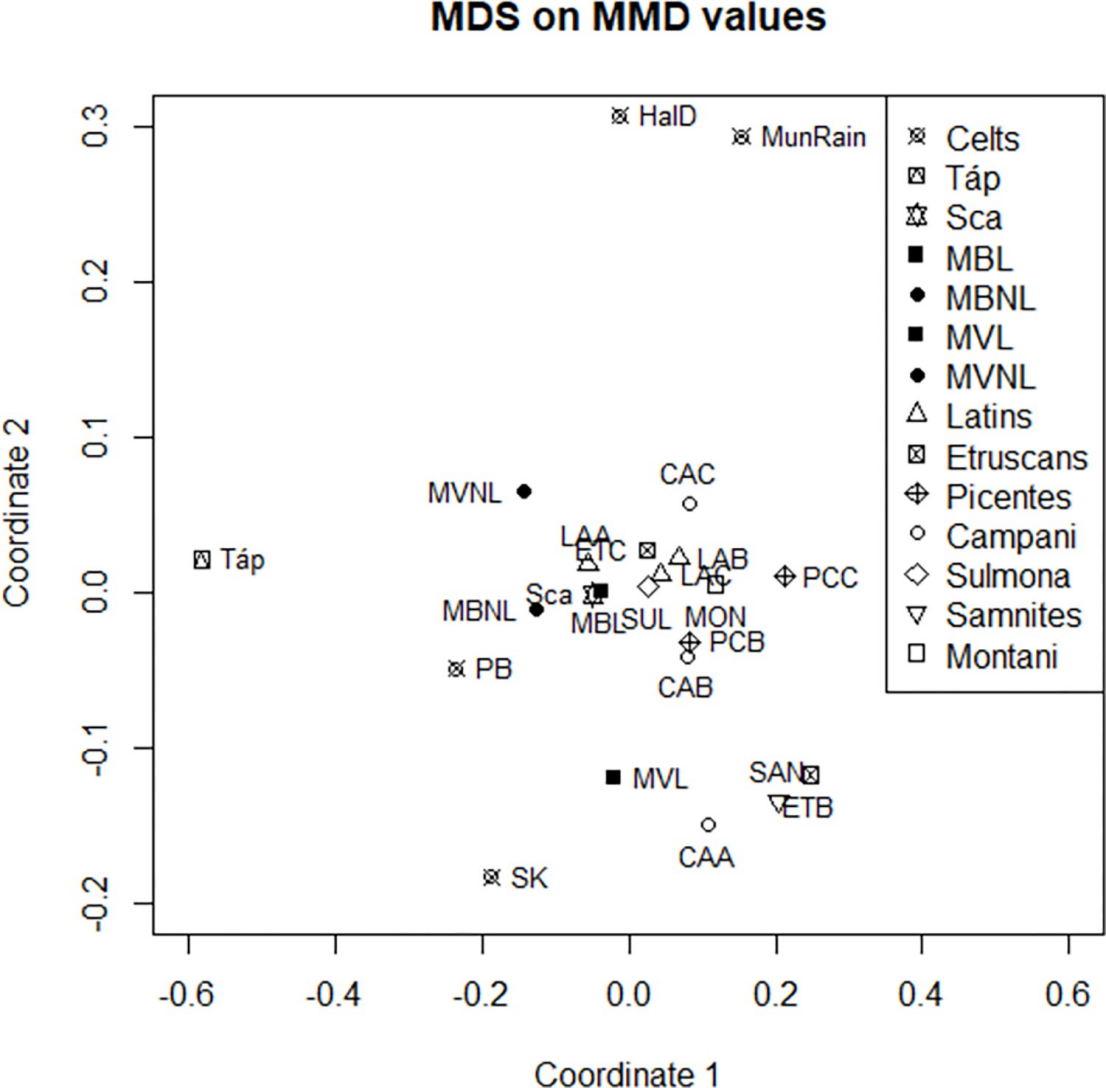
Two-dimensional MDS based on MMD values.

**Fig 4:**
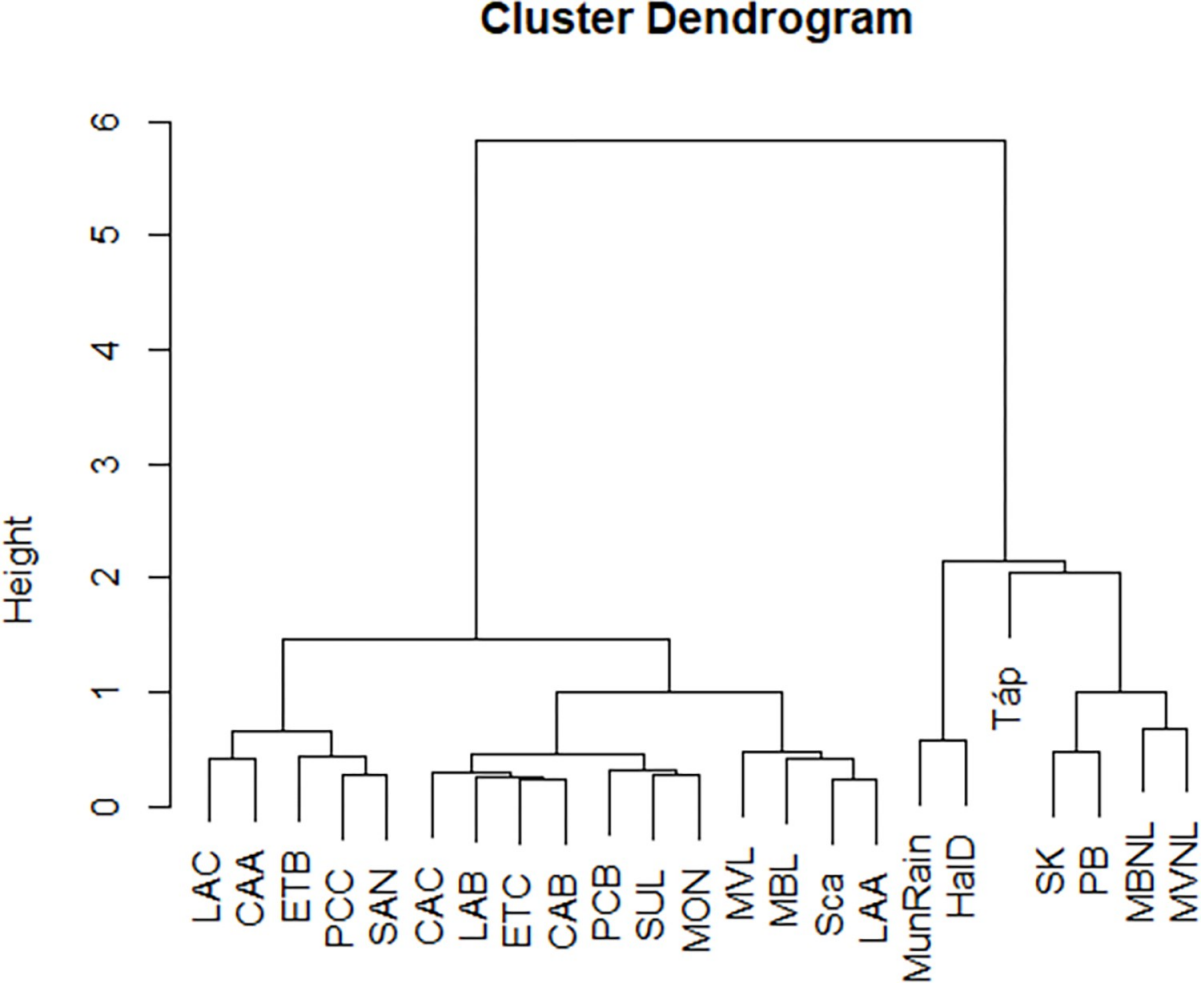
Cluster dendrogram based on MMD values.

Pairwise MMD values and standard deviations (SD) are reported in Table 4. MMD values significance based on the relationship MMD>2SD is shown in S3 Table. Among all the non-metric dental traits considered in this analysis, the lower second premolar lingual cusp number (Lingual Cusp LP2) presented with the highest power in discriminating between populations (Table 5). Biological distance among groups as shown by MDS (Fig 3) and cluster dendrogram (Fig 4) confirms results previously reported by Sorrentino and colleagues [4]. The MDS plot (Fig 3) shows a clear separation between Celts and Italics (significant MMD range of 0.122–0.561), with Italics more closely clustered at the centre of the graph, Celts more dispersed on a scattered line (significant MMD range of 0.201–0.582) to the left of the Italic cluster, and Scythians-related sample falling far from both groups but closer to Celts (significant MMD range of 0.608–0.695) than to Italics (significant MMD range of 0.271–0.830).

**Table 4:** MMD value (above) and SD (below) for 10 dental traits among the sample^1^.

	SK	PB	Táp	Sca	MBL	MBNL	MVL	MVNL	Mun Rain	HalD	LAA	LAB	LAC	ETB	ETC	PCB	PCC	CAA	CAB	CAC	SUL	SAN	MON
**SK**		0.000	0.000	0.160	**0.265**	**0.403**	0.047	0.000	**0.582**	**0.487**	0.103	**0.249**	**0.311**	**0.270**	**0.170**	**0.179**	**0.374**	**0.218**	0.163	**0.314**	**0.189**	**0.353**	**0.298**
**PB**	0.122		0.119	**0.253**	**0.162**	**0.252**	0.034	0.000	**0.546**	**0.397**	**0.251**	**0.291**	**0.306**	**0.391**	**0.198**	**0.369**	**0.456**	**0.457**	**0.251**	**0.228**	**0.280**	**0.421**	**0.289**
**Táp**	0.161	0.122		0.302	**0.381**	0.277	**0.455**	0.072	**0.695**	**0.608**	**0.271**	**0.574**	**0.525**	**0.830**	**0.500**	**0.591**	**0.769**	**0.652**	**0.625**	**0.604**	**0.475**	**0.777**	**0.644**
**Sca**	0.142	0.103	0.143		0.000	0.000	0.000	0.070	**0.278**	**0.270**	0.000	0.000	0.000	0.133	0.004	0.035	0.005	0.009	0.000	0.086	0.000	0.026	0.022
**MBL**	0.114	0.072	0.113	0.095		0.000	0.032	0.038	**0.298**	**0.311**	0.119	0.020	**0.111**	**0.182**	0.029	**0.137**	**0.140**	**0.141**	0.064	0.019	0.025	**0.088**	**0.116**
**MBNL**	0.176	0.137	0.178	0.159	0.129		0.116	0.051	**0.521**	**0.337**	0.096	0.094	**0.271**	**0.448**	**0.170**	**0.260**	**0.214**	**0.258**	0.121	**0.122**	**0.262**	**0.207**	**0.315**
**MVL**	0.148	0.109	0.149	0.132	0.101	0.167		0.005	**0.409**	**0.434**	0.000	0.000	0.047	0.084	0.000	0.056	0.018	0.116	0.000	0.050	0.056	0.000	0.000
**MVNL**	0.141	0.098	0.138	0.122	0.091	0.155	0.129		**0.221**	**0.201**	0.000	0.188	**0.255**	**0.348**	0.060	0.142	**0.207**	**0.252**	0.077	0.092	0.157	**0.225**	**0.223**
**MunRain**	0.096	0.054	0.094	0.077	0.045	0.112	0.083	0.073		**0.245**	**0.225**	**0.251**	**0.267**	**0.392**	**0.173**	**0.283**	**0.249**	**0.484**	**0.292**	**0.151**	**0.170**	**0.361**	**0.169**
**HalD**	0.097	0.055	0.096	0.078	0.047	0.113	0.084	0.074	0.028		**0.241**	**0.196**	**0.321**	**0.460**	**0.225**	**0.350**	**0.340**	**0.467**	**0.344**	**0.204**	**0.340**	**0.561**	**0.363**
**LAA**	0.125	0.084	0.126	0.107	0.076	0.142	0.113	0.102	0.057	0.059		0.000	0.063	0.127	0.000	0.000	0.019	0.062	0.000	0.048	0.095	0.088	0.049
**LAB**	0.125	0.086	0.127	0.109	0.077	0.141	0.114	0.103	0.059	0.061	0.090		0.004	0.000	0.000	0.000	0.000	0.025	0.000	0.000	0.000	0.000	0.000
**LAC**	0.132	0.102	0.139	0.117	0.089	0.155	0.122	0.110	0.075	0.076	0.103	0.101		**0.191**	0.090	0.123	0.115	0.057	0.139	**0.107**	0.142	0.176	0.040
**ETB**	0.113	0.073	0.115	0.095	0.065	0.130	0.102	0.091	0.047	0.048	0.077	0.081	0.089		0.000	0.005	0.071	**0.107**	0.000	0.035	0.012	**0.050**	0.000
**ETC**	0.104	0.062	0.103	0.085	0.053	0.119	0.091	0.081	0.035	0.036	0.066	0.069	0.080	0.057		0.000	0.004	**0.097**	0.000	0.000	0.000	0.021	0.000
**PCB**	0.093	0.051	0.091	0.074	0.042	0.108	0.080	0.069	0.023	0.024	0.054	0.056	0.072	0.044	0.032		0.020	0.014	0.000	0.039	0.051	**0.045**	0.049
**PCC**	0.090	0.047	0.088	0.070	0.038	0.105	0.076	0.066	0.020	0.021	0.051	0.053	0.070	0.041	0.029	0.016		**0.100**	0.000	**0.042**	**0.061**	0.017	0.013
**CAA**	0.105	0.063	0.104	0.086	0.054	0.121	0.092	0.082	0.036	0.037	0.067	0.069	0.082	0.057	0.045	0.033	0.029		0.065	**0.154**	0.167	**0.112**	**0.191**
**CAB**	0.122	0.081	0.122	0.105	0.073	0.139	0.112	0.101	0.056	0.057	0.086	0.088	0.095	0.076	0.065	0.053	0.049	0.065		0.000	0.000	0.000	0.000
**CAC**	0.102	0.062	0.102	0.084	0.052	0.118	0.090	0.079	0.034	0.035	0.064	0.067	0.082	0.054	0.042	0.030	0.027	0.043	0.063		0.043	**0.060**	0.000
**SUL**	0.113	0.070	0.110	0.094	0.062	0.127	0.100	0.090	0.044	0.045	0.074	0.077	0.085	0.064	0.053	0.041	0.037	0.054	0.073	0.051		0.044	0.000
**SAN**	0.093	0.050	0.091	0.073	0.041	0.108	0.079	0.069	0.023	0.024	0.054	0.056	0.072	0.044	0.032	0.019	0.016	0.032	0.052	0.030	0.040		0.021
**MON**	0.108	0.066	0.108	0.090	0.058	0.123	0.095	0.085	0.040	0.041	0.070	0.073	0.084	0.061	0.049	0.037	0.033	0.049	0.069	0.047	0.057	0.036	

^1^ Bold values indicate statistical significance when MMD is greater than twice the standard deviation.

**Table 5:** Overall measure of divergence (MD) for each variable sorted in decreasing order of discriminatory power.

	Overall MD
Lingual Cusp LP2	97.413
Distal Accessory Ridge UC	55.525
Double shoveling UI1	51.789
Premolar root number UP1	46.634
Carabelli’s trait UM1	44.785
Tuberculum dentale UI2	39.323
Parastyle UM3	33.384
Molar root number_UM2	28.943
Groove Pattern LM2	19.194
Cusp Number LM1	2.067

Similarly, in the hierarchical clustering dendrogram (Fig 4), Italics and Celts cluster separately on two main different branches, with Scythians-related sample locating at the base of the branch shared between Celts from the Carpathian Basin and Italy, and continental Celts grouping separately from them. The local branch hosts all the Iron Age Italic and Bronze Age individuals, and there is a close proximity with the three northernmost samples (MBL, MVL and Sca).

## Discussion

Our data show a biological similarity among all Celtic groups (Figs 3 and 4), in respect to the other populations considered in this study, suggesting that the whole Celtic sample, comprising Celts from Hungary, Italy ([4] and this study), Austria and Switzerland [5] likely shares a common genetic background that emerges when the Celtic groups are compared to non-Celtic populations. This evidence ultimately supports the historical and archaeological records that would have seen a common origin for the individuals of the Celtic culture, although being aware that the Celtic koiné displayed regional differences [1, 5, 6, 15–17]. In fact, the MMD values reveal some significant differences among Celtic populations (Table 4) supporting the existence of regional heterogeneity. For the first time, we report biological data for the Iron Age necropolises of Sopron-Krautacker and Pilismarót-Basaharc from Hungary, whose Celtic genetic background is supported by the fact that they fall into the Celtic cluster (Fig 4) as well as by robust material cultural evidence [36, 38, 40, 41, 88–91, 93, 94]. The analysis of strontium isotopes ([3] and this study; Fig 2 and Table 3) and non-metric dental traits for the Etruscan-Celtic site of Monte Bibele confirms the non-local status (i.e. Celtic-like) of a group of individuals from this necropolis (MBNL) and its biological affinity with the non-local individuals (likely Celts) from the close and coeval necropolis of Monterenzio Vecchio (MVNL; [4]). Historical records mentioning a common origin for Celts migrated southwards and eastwards from their core region in Western-central Europe, and more specifically Gaul (Ius., XXIV, 4; Tit. Liv., V, 34) may find support in the biological affinity between Celts from the Carpathian Basin (SK and PB) and Italian non-local individuals (i.e. Celtic-like) from Monte Bibele and Monterenzio Vecchio (MBNL and MVNL).

Furthermore, non-metric dental results (Fig 4) showed a biological proximity of the Scythians-related sample (Táp; this study) with Celts from the Carpathian Basin (SK and PB) and non-locals from Italy (MBNL and MVNL), suggesting that these two groups could share a similar ancestry. Archaeologists argue in favour that the historical Scythians appeared in the Iron Age and their core origin area was settled in the Eurasian Steppe [135], as recently explored from a genetic point of view [136, 137]. Although being aware that during the Iron Age an inflow of Steppean cultural and genetic impact came to the Carpathian Basin [49], it is nevertheless important to remember that Scythians-related populations in the Carpathian Basin have a very strong local Late Bronze Age background [48].

The supposed shared ancestry of the Iron Age Scythians-related population and Celts from the Carpathian Basin and the Italian peninsula may be likely traced back to a common biological background that was present in the Eurasian Steppe during the Bronze Age, allowing to consider this one as a key period to explore the genetic background of migrants who contributed to the diversity of the Iron Age genic pool in Italy and the Carpathian Basin, but further studies are needed to corroborate this hypothesis. Therefore, this study is a hint of a likely non-Celtic (i.e., Steppean) component with a plausible Bronze Age origin in the Carpathian and Italian groups attributed archeologically, and now biologically as well, to the Celtic culture. This agrees with the recent studies that highlighted a westward migration of individuals from the Eurasian Steppe towards Italy during Bronze Age [138, 139]. It is likely that a migration route would have first interested the Carpathian Basin during the Bronze Age, contributing later to the genic pool of descendant populations living in Hungary during the Iron Age, like the Scythians-related necropolis of Tápiószele and the Celts from Sopron-Krautacker and Pilismarót-Basaharc. Further, a westward migratory route could have brought people from the Carpathian Basin to the Italian peninsula, here establishing an intake of eastern genes which could have merged with the amount of biological variability that was under rearrangement in Europe due to the Celtic migrations during Iron Age. This hypothesis is supported by observing the Celtic cluster (Fig 4), where the branch comprising Scythians-related population, Celts from the Carpathian Basin and Italian Celts is clearly separated from the branch hosting Celtic populations from continental regions (Austria, and Switzerland [5]). The latters are here supposed to have a lack of marginal interaction with the abovementioned westward route from the Eurasian Steppe to Italy first proposed by Posth and colleagues [139]. The aforementioned difference between the group of Scythians-related population, Celts from the Carpathian Basin and Italian Celts compared to the group of Celtic populations from continental regions could be driven by the phenotype of the lower second premolar lingual cusp number (Lingual Cusp LP2), resulting to be the dental trait with the highest discriminatory power (Tab 5), contributing to set the distance between the former group, that showed the highest percentage of presence, compared to the latter one. This result could support our abovementioned hypothesis of the existence of biological contacts along the route between the Carpathian Basin and the Italian Peninsula.

On the other side, when focusing on the variability in the Italian peninsula, it is possible to detect the strong local (i.e. Italian) background of all the samples gathered in a single branch (Fig 4; [4, 62] and this study) although recognizing that the use of pooled samples (i.e., [62]) may not adequately represent the range of variation within the original populations, and that further samples could be useful to determine the biological affinity within the regions represented by pooled samples. Furthermore, we notice that the Iron Age local sample from Monte Bibele (MBL) clusters at its closest with the local individuals from Monterenzio Vecchio (MVL), again confirming the hypothesis of a strong biological similarity between these samples from two adjacent archaeological sites.

Due to the fact that a close branch is shared between our northernmost Iron Age local individuals (MBL and MVL) and the Bronze Age necropolis of Scalvinetto (Sca; this study), we can propose the existence, during Bronze and Iron Age, of lively migratory routes in northern Italy. This is also confirmed by the recent evidence that the settlement of Fondo Paviani, associated with the cemetery of Scalvinetto, was a place of interest for groups of migrants [87]. At the same time, we can support the persistence of a strong autochthonous pre-Iron Age substrate in later local communities (MBL and MVL) which undoubtedly witnessed the arrival of Celtic newcomers (MBNL and MVNL), and resulted in being the only example of cultural integration between Etruscans and Celts in Italy [30]. Even if the Etruscan-like (MBL and MVL) and Celtic-like individuals (MBNL and MVNL) are located on two different branches of the dendrogram, thus giving evidence of the biological differences between Celts and autochthonous inhabitants of northern Italy (Fig 4), we cannot exclude the possibility of intermarriage between these two ethnic groups in the Emilia-Romagna region. This hypothesis is also supported by the MDS plot (Fig 3), where the locals (MBL and MVL) are much closer to their Celtic cohabitants (MBNL and MVNL) than to the Celts of the core regions (HalD and MunRain; [5]).As shown by the MMD values (Table 4), the Celts residing in northern Italy show a diversified pattern of similarities and statistically significant differences with the Bronze Age populations, Iron Age Italics and other Celts. In particular, Italian Celts (MBNL and MVNL) do not show statistically significant differences with Scalvinetto and with northern Italian Etruscans (MBL and MVL). These results are an additional support to the hypotheses of biological and cultural admixture between Celts and Italics cohabiting in Monte Bibele and Monterenzio Vecchio [4, 97–103].

Moreover, it is relevant to note that the Bronze Age site of Scalvinetto, the oldest sample in our study, shares a biological affinity with one of the oldest groups of the Italic sample, which dates back to the beginning of the Iron Age (LAA, i.e., Ancient Latini; [62]), suggesting the existence of a common biological background for the autochthonous population of Italy that could be dated back to the Bronze Age.

Besides the fact that this study focused on the migratory routes during the Iron Age, and specifically the migratory patterns of Celts and their interactions with the local populations they encountered, our results highlight that most of our knowledge on the pre-existing biological and genetic background during the Bronze Age could have had a pivotal role in shaping the populations of the Iron Age in Europe. Future studies are needed to elucidate when and how migration routes crossed the Alps during the Bronze Age, determining biological networks spanned far from time and geography as highlighted in the present study. Furthermore, future research will be useful to determine if the patterns indicated in this study are also found in other regional populations.

## Conclusion

This research article supports the existence of flows of people during the Iron Age towards Eastern and Southern Europe. In the Iron Age, Celts spread extensively, reaching northern Italy and interacting with autochthonous Etruscans, and heading towards the Carpathian Basin where they had relationships with local groups such as the Scythians-related populations. Nevertheless, the persistence of regional variation among Celtic groups throughout Europe cannot be ruled out [1, 5, 6, 15–17]. Meanwhile, a westward flow of Steppe-related people, for which a Steppean related ancestry is highly supported, had probably already begun during the Bronze Age, leaving biological traces in the Carpathian Basin and later witnessing the Iron Age arrival of Western European Celts [5], that resulted in the prosecution towards Italy of a westward migration being enriched by Celtic variability. At the same time, our results strengthen the hypothesis of the presence of a solid local biological substrate in Italy dating back to the Bronze Age, that strongly survived in the Iron Age, nevertheless being interested by the admixture of an incoming variability due to Celtic flows.

## Supporting information

S1 Table Specimen numbers, repository information and sex and age information of the samples analyzed for the first time in this study. (XLSX)

S2 Table Percentages (%) of 42 discrete dental traits and number of individuals (n) in the samples before data cleaning. (XLSX)

S3 Table Matrix of significance for the MMD values based on the relationship MMD>2SD. (XLSX)

S1 Fig Pearson’s product-moment of correlation (below) and *p*-value (above) of the novel data. (TIF)
