# Correction: New insights on Celtic migration in Hungary and Italy through the analysis of non-metric dental traits

**DOI:** 10.1371/journal.pone.0316684

**Published:** 2024-12-26

**Authors:** Erica Piccirilli, Rita Sorrentino, Federico Lugli, Eugenio Bortolini, Sara Silvestrini, Claudio Cavazzuti, Sara Conti, Szabolcs Czifra, Katalin Gyenesei, Kitti Köhler, Károly Tankó, Antonino Vazzana, Erzsébet Jerem, Anna Cipriani, Antonio Gottarelli, Maria Giovanna Belcastro, Tamás Hajdu, Stefano Benazzi

The affiliation for the 13^th^ author is incorrect. The correct affiliation is not indicated. Erzsébet Jerem is not affiliated with #8 but with: HUN-REN Bölcsészettudományi Kutatóközpont Régészeti Intézet, HUN-REN Research Centre for the Humanities, Institute of Archaeology, Tóth Kálmán utca 4, H-1097, Budapest.
