# Supplementary figures and images for: New insights on Celtic migration in Hungary and Italy through the analysis of non-metric dental traits
Source: PLoS One. 2023 Oct 18;18(10):e0293090. doi: 10.1371/journal.pone.0293090 (PMC10584115; doi:10.1371/journal.pone.0293090)

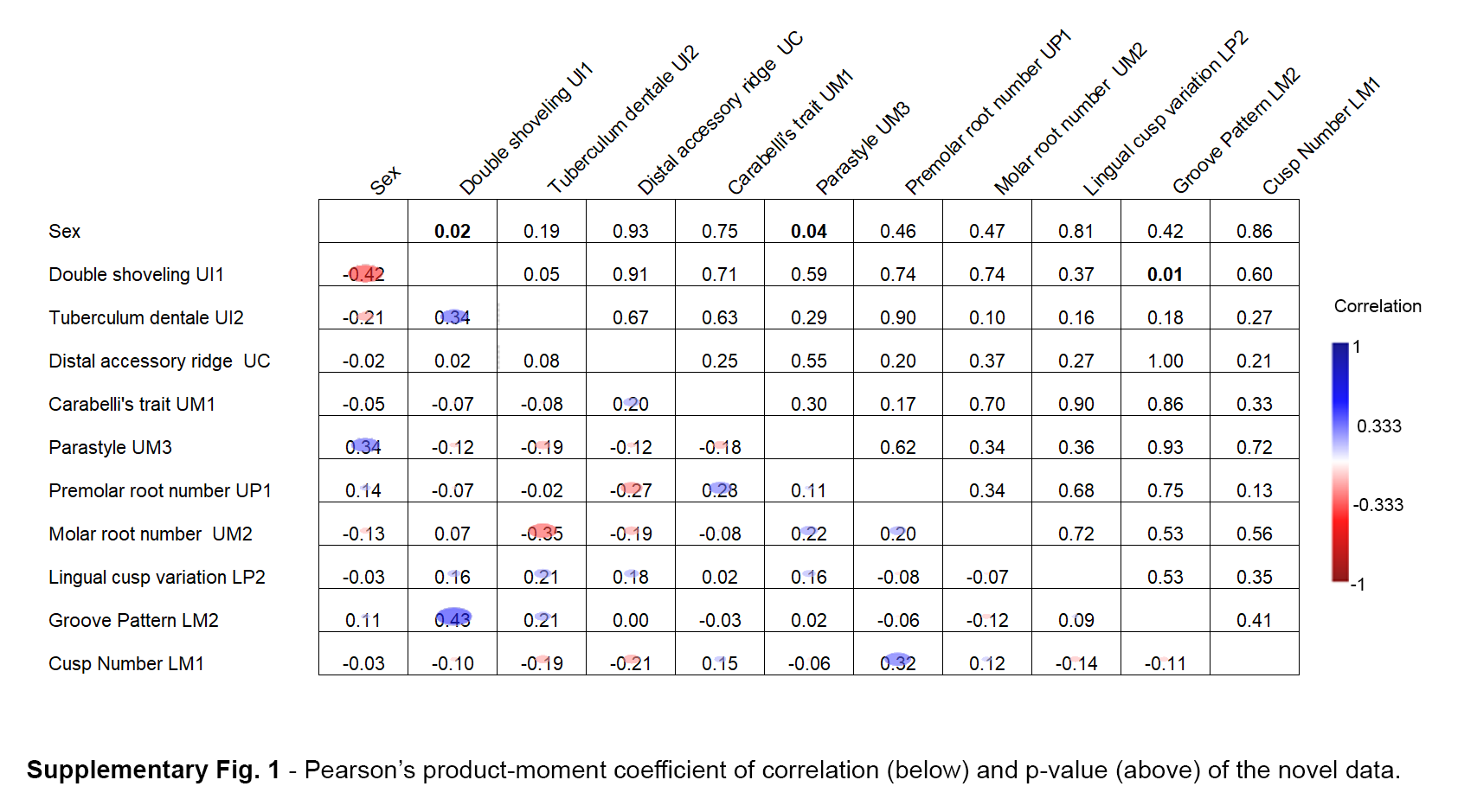

Supplement: S1 Fig — (TIF) [file pone.0293090.s004.tif]
